# Infrapatellar fat pad resection or preservation during total knee arthroplasty: a meta-analysis of randomized controlled trials

**DOI:** 10.1186/s13018-020-01823-2

**Published:** 2020-08-05

**Authors:** Changjiao Sun, Xiaofei Zhang, Woo Guan Lee, Yan Tu, Huimin Li, Xu Cai, Huadong Yang

**Affiliations:** 1 Department of Orthopedic, Beijing Tsinghua Changgung Hospital, School of Clinical Medicine, Tsinghua University, No.168 Litang Road, Dongxiaokou Town, Changping District, Beijing, 102218 China; 2 Department of Clinical Epidemiology and Biostatistics, Beijing Tsinghua Changgung Hospital, School of Clinical Medicine, Tsinghua University, No.168 Litang Road, Dongxiaokou Town, Changping District, Beijing, 102218 China; 3 Department of Orthopedic, Kuching Specialist Hospital, Tabuan Stutong Commercial Centre, 93350 Kuching, Sarawak Malaysia

**Keywords:** Total knee arthroplasty, Infrapatellar fat pad, Hoffa’s fat pad, patellar tendon length, Insall-Salvati ratio

## Abstract

**Background:**

The infrapatellar fat pad (IPFP) or Hoffa’s fat pad is often resected during total knee arthroplasty in order to improve visibility. However, the management of the IPFP during total knee arthroplasty (TKA) is the subject of an ongoing debate that has no clear consensus. The purpose of this review was to appraise if resection of the IPFP affects clinical outcomes.

**Methods:**

We conducted a meta-analysis to identify relevant randomized controlled trials involving infrapatellar fat pad resection and infrapatellar fat pad preservation during total knee arthroplasty in electronic databases, including Web of Science, Embase, PubMed, Cochrane Controlled Trials Register, Cochrane Library, Highwire, CBM, CNKI, VIP, and Wanfang database, up to March 2020.

**Results:**

Nine randomized controlled trials, involving 783 TKAs (722 patients), were included in the systematic review. Outcome measures included patellar tendon length (PTL), Insall-Salvati ratio (ISR), rate of anterior knee pain, Knee Society Scores (KSS), and knee range of motion. The meta-analysis identified a trend toward the shortening of the patellar tendon with IPFP resection at 6 months (*P* = 0.0001) and 1 year (*P* = 0.001). We found no statistical difference in ISR (*P* = 0.87), rate of anterior knee pain within 6 months (*p* = 0.45) and 1 year (*p* = 0.38), KSS at 1 year (*p* = 0.77), and knee range of motion within 6 months (*p* = 0.61) and 1 year (0.46).

**Conclusion:**

Based on the available level I evidence, we were unable to conclude that one surgical technique of IPFP can definitively be considered superior over the other. More adequately powered and better-designed randomized controlled trial (RCT) studies with long-term follow-up are required to produce evidence-based guidelines regarding IPFP resection.

## Introduction

The infrapatellar fat pad (IPFP), also known as Hoffa’s fat pad, is located in the anterior compartment of the knee between the joint capsule and synovium. Often, it is resected during total knee arthroplasty procedures to allow for better visualization during the surgeon’s approach [1]. Resection of the IPFP is estimated to occur in around 88% of total knee arthroplasties (TKAs) [2]. However, the consequences of IPFP resection are the subject of an ongoing debate that has no clear consensus. There is mixed evidence regarding the risk of avascular necrosis following resection, with some studies suggesting compromising the blood supply to the patella leads to shortening of the patella tendon resulting in patella fracture [3, 4]. Moreover, others indicate it does not negatively affect the blood supply [5, 6]. Some studies indicate that removal of the fat pad could lead to increased post-operative stiffness [7, 8], while others suggest that there are no functional differences when comparing resection to preservation [9, 10]. The IPFP is thought to play a role in the inflammatory process and contains nociceptive fibers and is a possible source of anterior knee pain [11, 12]. Therefore, preservation of the IPFP may explain an increased incidence of anterior knee pain beyond 6 months post-operatively [13]. Meanwhile, Ioan-Facsinay and Kloppenburg [14] found that increased pain in patients with IPFP resection, remaining damaged nerve fibers may also be the source of ongoing pain in the IPFP resection group.

Given knowledge about how different management of the IPFP affects patient outcomes is variable, further information regarding how to handle the IPFP during TKA is necessary. Thus, this meta-analysis aims to objectively evaluate the influence of IPFP resection and preservation on TKA patient outcomes; we conducted a meta-analysis of current published evidence.

## Methods

The current meta-analysis was registered on PROSPERO (International Prospective Register of Systematic Reviews) and the registration number was CRD42020168616. The Cochrane Handbook for Systematic Reviews of Interventions PRISMA (Preferred Reporting Items for Systematic Reviews and Meta-analyses) guidelines were applied to assess the quality of the results published in all included studies to make sure the results of our meta-analysis reliable and veritable.

### Search strategy

We conducted a meta-analysis to identify relevant randomized controlled trials involving IPFP resection and preservation technique in total knee arthroplasty in electronic databases, including Web of Science, Embase, PubMed, Cochrane Controlled Trials Register, Cochrane Library, Highwire, CBM, CNKI, VIP, and Wanfang database, up to March 2020. The keywords used were “total knee arthroplasty,” “total knee replacement,” “infrapatellar fat pad (IPFP) or Hoffa’s fat pad,” “Hoffa’s fat pad,” “IPFP” in conjunction with Boolean operators “AND” or “OR.” Review Manager Software was used to perform the meta-analysis.

### Inclusion criteria

We included the study if it met the following inclusion criteria: (1) the intervention was the IPFP resection technique in TKA; (2) the comparator was the IPFP preservation technique; (3) the study designs were randomized controlled trial studies; (4) the outcomes were patellar tendon length (PTL), Insall-Salvati ratio (ISR), rate of anterior knee pain (AKP), Knee Society Scores (KSS), and knee range of motion (ROM); (5) the included studies were required to contain at least one outcome; and (6) the studies must have had a follow-up rate of at least 80%.

The exclusion criteria were as follows: (1) observational studies, (2) non-RCTs, and (3) studies with insufficient outcome data.

### Data extraction process

Two authors independently extracted the available data from each study. Disagreements were resolved by discussion to reach consensus. We extracted the primary data based on the following: first author, year of publication, number of TKAs and participants, age, gender, primary indication for TKA, follow-up time, primary outcome, prothesis, and patellar resurfacing. The primary outcome consisted of PTL, ISR, rate of AKP, and KSS. Secondary outcomes included knee ROM.

### Assessment of studies

According to the Cochrane Handbook for Systematic Reviews of Interventions, the methodological quality and basis of the included studies were assessed as follows: randomization, allocation concealment, blind method, selective reporting, group similarity at baseline, incomplete outcome data, compliance, timing of outcome assessments, and intention-to-treat analysis.

### Statistical analysis

We use Review Manager Software for MAC (version 5.3) to perform the meta-analysis. The chi-square was used to assess the significance of heterogeneity. *I*^2^ value> 50% suggested a high degree of heterogeneity; thus, we used the randomized-effects model. Otherwise, we used the fixed-effects model. The mean difference (MD) or standard MD was used to assess continuous outcomes such as PTL, ISR, KSS, and knee ROM with a 95% confidence interval (CI). Relative risks with a 95% CI were used to assess dichotomous outcomes such as the rate of AKP. If *P* values were less than 0.05, we considered the results as a statistically significant difference.

## Results

### Search results

**Fig. 1:**
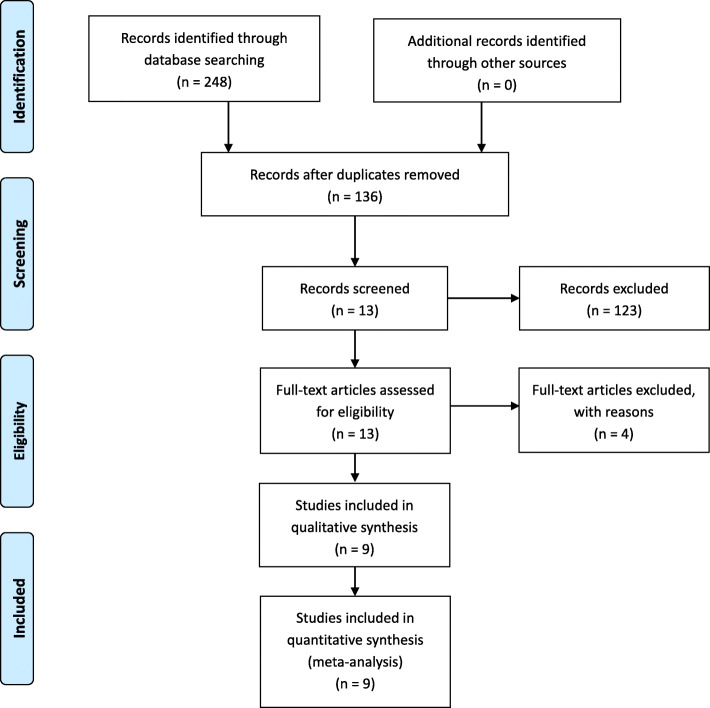
The search results and selection procedure. Legend: The literature search identified 248 citations. Of these, 112 duplicates were removed. Upon review of titles and abstracts of the 136 remaining articles, we excluded 123 papers according to the inclusion criteria; the full text of 13 articles was retrieved. Because sufficient data were not available in one article, three studies were non-RCTs; hence, four studies were excluded. Finally, we identified 783 TKAs (722 patients) assessed in 9 randomized controlled trials

The literature search and selection process are shown in Fig. 1. The literature search identified 248 citations. Of these, 112 duplicates were removed. Upon review of titles and abstracts of the 136 remaining articles, we excluded 123 papers according to the inclusion criteria; the full text of 13 articles was retrieved. Because sufficient data were not available in one article, three studies were non-RCTs; hence, four studies were excluded. Finally, we identified 783 TKAs (722 patients) assessed in 9 randomized controlled trials [1, 9, 15–20]. We presented the detailed baseline characteristics and general intervention information in Table 1. All the articles were published in English and Chinese between the years 2003 and March 2020.

**Table 1:** The detailed baseline characteristics and general intervention information

Study	Infrapatellar fat pad resection/infrapatellar fat pad preservation								
	TKAs	Patients	Age (years)	Female gender (%)	Primary indication for TKA	Mean follow-up time	Primary outcome	Prothesis	Patellar resurfacing
Lemon et al. [1]	35/38	35/38	73.8/71.5	65.7/65.8	Osteoarthritis	3 year	(1) Patellar tendon length	Uncemented prosthesis (Profix, Smith and Nephew, Memphis, TN, USA)	No
Pinsornsak et al. [9]	36/41	36/41	67.1/68.2	97.2/90.2	Osteoarthritis and rheumatoid arthritis	1 year	(1) Rate of anterior knee pain	NA	NA
Maculé et al. [10]	34/34	34/34	NA	NA	Osteoarthritis	6 month	(1) Patellar tendon length,(2) rate of anterior knee pain	CR (Profix®, Smith and Nephew, Memphis, USA) and (Scorpio®, Stryker, Mahwah, USA)	No
Tanaka et al. [15]	54/53	40/40	54.4/53.7	72.5/77.5	rheumatoid arthritis	3 year	(1) Rate of anterior knee pain,(2) range of motion,(3) patellar tendon length difference	NA	Yes
Li et al. [16]	38/38	38/38	65.25/65.16	60.5/63.2	Osteoarthritis	1 year	(1) Range of motion,(2) Insall-Salvati ratio (ISR), (3) blood loss,(4) operation time	NA	NA
Xin et al. [17]	33/33	33/33	70.12	NA	Osteoarthritis	1 year	(1) Insall-Salvati ratio (ISR), (2) patellar tendon length	PS	No
Li et al. [18]	51/53	39/39	67/68	66.7/71.8	Osteoarthritis	6 month	(1) Range of motion,(2) rate of anterior knee pain,(3) blood loss,(4) operation time,(5) patellar tendon length	NA	No
Zhou et al. [19]	59/59	110	68/67.6	71.2/64.4	Osteoarthritis	1 year	(1) Insall-Salvati ratio (ISR), (2) KSS	Genesis II⁃PS (Smith and Nephew)	No
Liu et al. [20]	48/46	48/46	62.9/61.83	85.4/80.4	Osteoarthritis	1 year	(1) Range of motion,(2) KSS(3), patellar tendon length	NA	No

The detailed baseline characteristics information including the number of TKAs, age, gender, primary indication of TKA, mean follow-up time, primary outcome, prothesis, and patellar resurfacing of two groups

### Risk of bias assessment

**Fig. 2:**
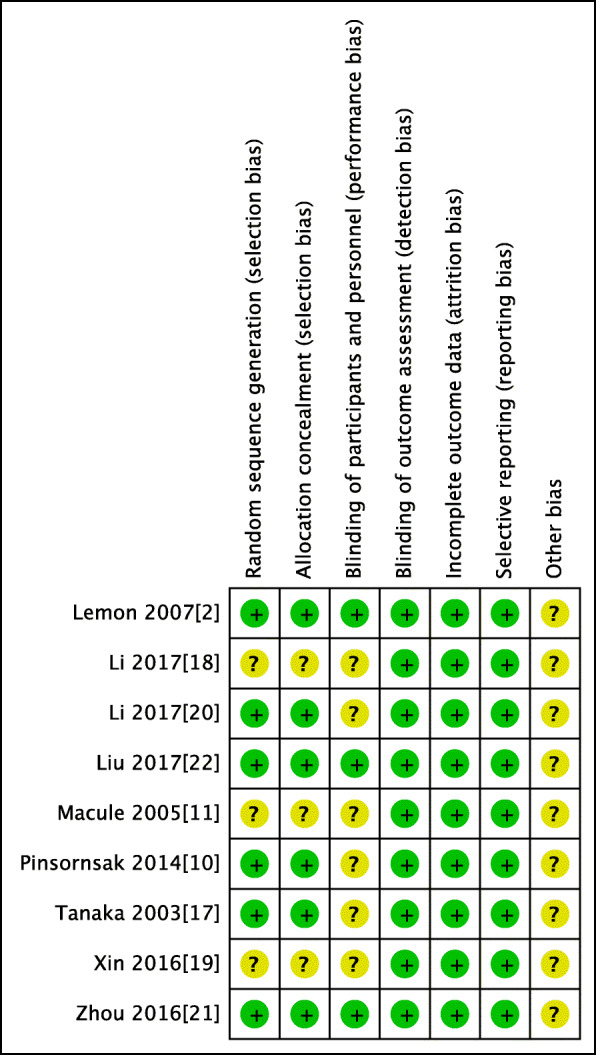
Risk of bias included in the randomized controlled trials. Legend: Plus sign indicates no bias; hyphen, bias; and question mark, bias unknown. Six studies adequately described the correct randomization and sufficient allocation concealment. Nine studies described the blinding of outcome assessment, and three studies described the blinding of participants and personnel. All studies retained complete outcome data, avoided selective reporting, and had an unclear risk of other bias

**Fig. 3:**
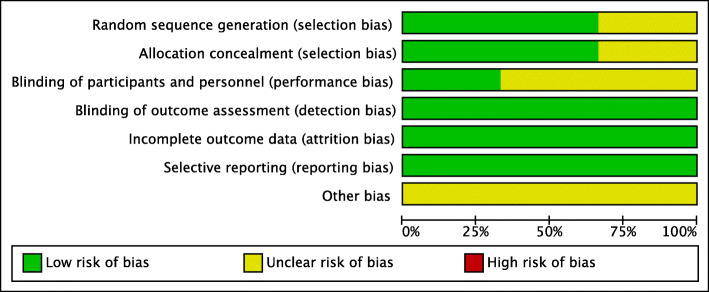
The risk of bias graph. Legend: The overall quality of the included studies was considered adequate

The risk of bias summary and risk of bias graph for RCTs are shown in Figs. 2 and 3, respectively. Six studies adequately described the correct randomization and sufficient allocation concealment. Nine studies described the blinding of outcome assessment, and three studies described the blinding of participants and personnel. All studies retained complete outcome data and avoided selective reporting. We cannot ignore other potential risks of biases of all studies. Therefore, we rated as having an unclear risk of other bias. As a result, the overall quality of the included studies was considered adequate (Figs. 2 and 3).

### Pooled analysis of PTL between IPFP resection and IPFP preservation

**Fig. 4:**
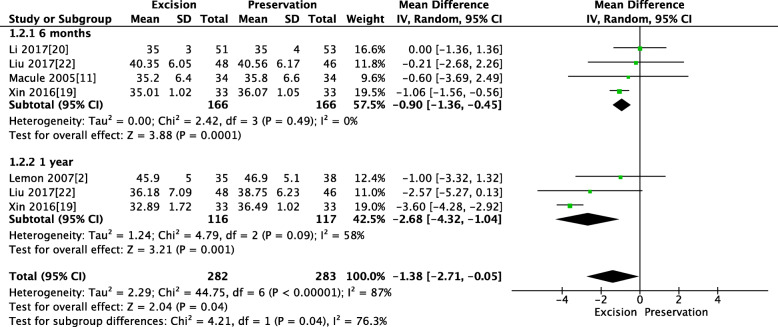
Pooled analysis of PTL between IPFP resection and IPFP preservation. Legend: Four RCTs reported the PTL at 6 months, and three RCTs reported PTL at 1 year. The pooled results showed that a trend toward shortening of the patellar tendon with IPFP resection at 6 months (MD − 0.9, 95% CI [− 1.36, − 0.45], *P* = 0.0001) and 1 year (MD − 2.68, 95% CI [− 4.32, − 1.04], *P* = 0.001)

Four RCTs reported the PTL at 6 months, and three RCTs reported PTL at 1 year. The pooled results showed that a trend toward shortening of the patellar tendon with IPFP resection at 6 months (MD − 0.9, 95% CI [− 1.36, − 0.45], *P* = 0.0001; Fig. 4) and 1 year (MD − 2.68, 95% CI [− 4.32, − 1.04], P = 0.001; Fig. 4). This finding was reported to be statistically significant.

### Pooled analysis of ISR between IPFP resection and IPFP preservation

**Fig. 5:**
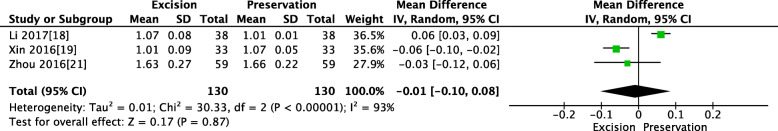
*P* pooled analysis of ISR between IPFP resection and IPFP preservation. Legend: Three studies reported the ISR; we found some statistical heterogeneity between the two groups of ISR (*x*^2^ = 30.33; df = 2, *P* < 0.00001; *I*^2^ = 93%); thus, a random-effects model was used. The pooled results showed that patients in both groups experienced similar ISR (MD = − 0.03, 95% CI [− 0.12, 0.08], *P* = 0.87)

Three studies reported the ISR; we found some statistical heterogeneity between the two groups of ISR (*x*^2^ = 30.33; df = 2, *P* < 0.00001; *I*^2^ = 93%; Fig. 5); thus, a random-effects model was used. The pooled results showed that patients in both groups experienced similar ISR (MD = − 0.03, 95% CI [− 0.12, 0.08], *P* = 0.87; Fig. 5).

### Pooled analysis of the rate of anterior knee pain between IPFP resection and IPFP

**Fig. 6:**
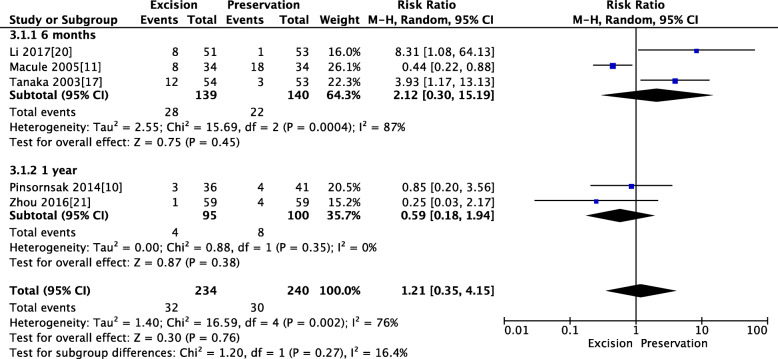
Pooled analysis of the rate of anterior knee pain between IPFP resection and IPFP. Legend: Three RCTs reported the rate of anterior knee pain within 6 months, and two RCTs reported the rate of anterior knee pain at 1 year. The pooled results showed that patients in both groups experienced similar rates of anterior knee pain within 6 months (MD 2.12, 95% CI [0.3, 15.19], *P* = 0.45) and 1 year (MD 0.59, 95% CI [0.18, 1.94], *P* = 0.38)

Three RCTs reported the rate of anterior knee pain within 6 months, and two RCTs reported the rate of anterior knee pain at 1 year. The pooled results showed that patients in both groups experienced similar rates of anterior knee pain within 6 months (MD 2.12, 95% CI [0.3, 15.19], *P* = 0.45; Fig. 6) and 1 year (MD 0.59, 95% CI [0.18, 1.94], *P* = 0.38; Fig. 6)

### Pooled analysis of KSS between IPFP resection and IPFP preservation

**Fig. 7:**
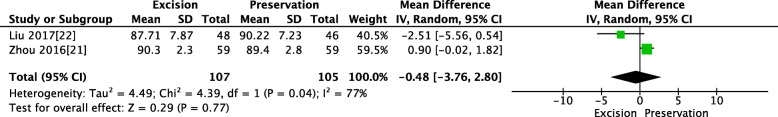
Pooled analysis of KSS between IPFP resection and IPFP preservation. Legend: Two studies reported KSS at 1 year; we found some statistical heterogeneity between the two groups (*x*^2^ = 4.49; df = 1, *P* = 0.04; *I*^2^ = 77%), and thus a random-effects model was used. The pooled results showed that patients in both groups experienced similar KSS (MD = − 0.48, 95% CI [− 3.76, 2.80], *P* = 0.77)

Two studies reported KSS at 1 year; we found some statistical heterogeneity between the two groups (*x*^2^ = 4.49; df = 1, *P* = 0.04; *I*^2^ = 77%; Fig. 7), and thus a random-effects model was used. The pooled results showed that patients in both groups experienced similar KSS (MD = − 0.48, 95% CI [− 3.76, 2.80], P = 0.77; Fig. 7).

### Pooled analysis of knee range of movements between IPFP resection and IPFP preservation

**Fig. 8:**
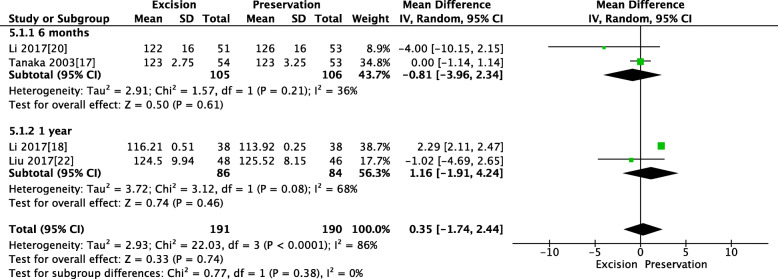
Pooled analysis of knee range of movements between IPFP resection and IPFP preservation. Legend: Two studies reported a knee range of movements within 6 months, and two studies reported a knee range of movements in 1 year. The pooled results showed that patients in both groups experienced a similar knee range of movements within 6 months (MD = − 0.81, 95% CI [− 3.96, 2.34], *P* = 0.61) and at 1 year (MD = 1.16, 95% CI [− 1.91, 4.24], *P* = 0.46)

Two studies reported a knee range of movements within 6 months, and two studies reported a knee range of movements in 1 year. The pooled results showed that patients in both groups experienced a similar knee range of movements within 6 months (MD = − 0.81, 95% CI [− 3.96, 2.34], *P* = 0.61; Fig. 8) and at 1 year (MD = 1.16, 95% CI [− 1.91, 4.24], *P* = 0.46; Fig. 8).

## Discussion

This meta-analysis included nine RCTs that assessed 783 TKAs (722 patients) and directly compared the clinical effectiveness of IPFP resection and IPFP preservation. The pooled data indicated no difference between the two operation modes in terms of ISR, Rate of AKP within 6 months and at 1 year, KSS at 1 year and knee ROM within 6 months and at 1 year. But there is a trend toward shortening of the patellar tendon with IPFP resection at 6 months and 1 year. There are currently no formal national guidelines about IPFP resection or preservation during TKA. To our knowledge, this is the first meta-analysis of only RCTs comparing IPFP resection and IPFP preservation in primary TKA. We only found one meta-analysis [21] comparing IPFP resection and IPFP preservation in primary TKA. However, the inclusion criteria of the previous meta-analyses included only 2 RCTs and five retrospective cohort studies design, which are potentially subject to selection bias.

Furthermore, the restriction of the previous meta-analyses to English language publications potentially limits the power that could be obtained with the inclusion of patient enrollment from non-English language studies. Finally, they did not analyze the ISR and knee range of motion. Thus, based on the current studies comparing IPFP resection and IPFP preservation in TKA, we only included level I trials (RCTs) in our meta-analysis, which may have a more convincing persuasive result. We include not only English studies but also Chinese RCTs. We have different results in terms of pain between our meta-analysis and previous meta-analysis. Moreover, we included ISR and knee range of motion, which would provide a more exact conclusion and could be a supplement for the previous meta-analysis.

The IPFP has been shown to contain peptidergic C- and substance P positive nerve fibers, which is thought to play a role in the inflammatory process and therefore is a possible source of anterior knee pain [11–13]. Meanwhile, the remaining damaged nerve fibers were also the source of ongoing pain in the IPFP Resection group. The meta-analysis by Nisar et al. [21] demonstrated a trend toward increased pain in the early 1- to 2-month post-operative period in the IPFP-P group. At 3 to 6 months, this trend is reversed with a higher incidence of knee pain in the IPFP-R group. However, this trend is not supported by the present meta-analysis. In our meta-analysis, there is no statistical difference in the rate of anterior knee pain within 6 months and at 1 year. We think the change is likely due to seven recent RCTs providing new outcome data.

Our study showed a trend toward patellar tendon shortening in the IPFP resection group, which agreed with Nisar et al.'s result. When the IPFP is damaged, it undergoes fibrosclerotic change. Fibrous tissue bands pass through this area and create a non-extensile, rigid structure [22]. This, therefore, leads to the shortening of the patellar tendon. Some studies have shown that shortening of the patella tendon may result in a patella fracture [3, 4]. However, no patella fracture occurred in all our IPFP resection groups.

ISR was one of the new findings of our meta-analysis compared to past meta-analyses. Our study has shown that ISR was not significantly changed following IPFP excision, which seems to contradict findings indicating that PTL was significantly shortened by resection in our study. This may be explained by the fact that the ISR index may not be a reliable parameter for post-operative TKA because osteophyte removal around the patellar bone affects the patellar bone length. However, other parameters (Blackburne-Peel ratio [23] and Caton-Deschamps [24]) are even less reliable for such measurements post-operatively because the joint line may change after TKA, which affects these parameters that required a consistent joint line for accurate evaluation [25].

Knee range of motion was another new finding of our meta-analysis compared to past meta-analyses. Tanaka et al.’s study reported a statistical decrease in flexion for the resection cohort [15]. However, there was evidence in our study suggesting that resection of the IPFP did not significantly affect knee flexion.

The utilization of the KSS was reported for the evaluation of post-operative function in three studies. There was no difference found for our study comparing post-operative KSS scores between patients undergoing resection and those with IPFP preservation which agreed with the previous meta-analysis

To the best of our knowledge, this is the first meta-analysis comparing outcomes between IPFP resection and IPFP preservation for TKA that only includes randomized controlled trials. However, it has the following limitations. First, the data used in this study was derived from several studies evaluating different surgical techniques by different surgeons. As such, the technique and surgeons, although similar, were not identical. Second, while we did not observe any publication bias, we acknowledge, as others have, that results should be treated with caution when meta-analyses are based on a limited number of small trials, as is the case in the current investigation. Third, conducting an actual RCT is difficult. Only nine studies were RCTs, and the blinding of participants and personnel in the studies was difficult. Some papers did not report all of our outcomes of interest.

There are several strengths of this study that warrant mention: (1) Compared with the previous meta-analysis, we included some new clinical research up to March 2020; our results are therefore more up to date. (2) All included studies are RCTs which directly compared IPFP R and IPFP P. (3) Reporting was conducted on many new outcome measures such as range of motion and ISR which could be a supplement for the previous meta-analysis. (4) Some outcomes were sub-analyzed by categories of follow-up time.

## Conclusion

Based on the available level I evidence, we are unable to conclude whether resection or preservation should be the better operative choice. So we advised that surgeons could keep the fat pad if excellent exposure can be achieved but resect it if needed to improve exposure during TKA. More evidence is needed before one surgical technique can be definitively considered superior. We required more adequately powered and better-designed RCT studies with long-term follow-up to reach a firmer conclusion.

## Data Availability

The datasets generated and analyzed during the current study are available from the corresponding author on reasonable request.

## Abbreviations

CIs: Confidence intervals
RCTs: Randomized controlled trials
TKA: Total knee arthroplasty
IPFP: Infrapatellar fat pad
PTL: Patellar tendon length
ISR: Insall-Salvati ratio
AKP: Anterior knee pain
KSS: Knee Society Scores
ROM: Range of motion
PRISMA: Preferred Reporting Items for Systematic Reviews and Meta-analyses
